# Genome-wide identification and expression analysis of the U-box E3 ubiquitin ligase gene family related to bacterial wilt resistance in tobacco (*Nicotiana tabacum* L.) and eggplant (*Solanum melongena* L.)

**DOI:** 10.3389/fpls.2024.1425651

**Published:** 2024-07-30

**Authors:** Rui Chen, Gang Gu, Binghui Zhang, Chaofan Du, Xiaolu Lin, Weiwei Cai, Yan Zheng, Tong Li, Ruiqi Wang, Xiaofang Xie

**Affiliations:** ^1^ College of Life Sciences, Fujian Agriculture & Forestry University, Fuzhou, China; ^2^ Institute of Tobacco Science, Fujian Provincial Tobacco Company, Fuzhou, China; ^3^ Longyan Tobacco Company, Longyan, China; ^4^ Fujian Key Laboratory of Crop Breeding by Design, Fujian Agriculture & Forestry University, Fuzhou, China

**Keywords:** *U-box*, biotic stress, expression analysis, phylogenetic analysis, *Nicotiana tabacum *L., *Solanum melongena* L.

## Abstract

The E3 enzyme in the UPS pathway is a crucial factor for inhibiting substrate specificity. In Solanaceae, the U-box E3 ubiquitin ligase has a complex relationship with plant growth and development, and plays a pivotal role in responding to various biotic and abiotic stresses. The analysis of the *U-box* gene family in Solanaceae and its expression profile under different stresses holds significant implications. A total of 116 tobacco *NtU-boxs* and 56 eggplant *SmU-boxs* were identified based on their respective genome sequences. Phylogenetic analysis of *U-box* genes in tobacco, eggplant, tomato, *Arabidopsis*, pepper, and potato revealed five distinct subgroups (I-V). Gene structure and protein motifs analysis found a high degree of conservation in both exon/intron organization and protein motifs among tobacco and eggplant *U-box* genes especially the members within the same subfamily. A total of 15 pairs of segmental duplication and 1 gene pair of tandem duplication were identified in tobacco based on the analysis of gene duplication events, while 10 pairs of segmental duplication in eggplant. It is speculated that segmental duplication events are the primary driver for the expansion of the *U-box* gene family in both tobacco and eggplant. The promoters of *NtU-box* and *SmU-box* genes contained *cis*-regulatory elements associated with cellular development, phytohormones, environment stress, and photoresponsive elements. Transcriptomic data analysis shows that the expression levels of the tobacco and eggplant *U-box* genes in different tissues and various abiotic stress conditions. Using cultivar Hongda of tobacco and cultivar Yanzhi of eggplant as materials, qRT-PCR analysis has revealed that 15 selected *NtU-box* genes and 8 *SmU-box* may play important roles in response to pathogen *Ras* invasion both in tobacco and eggplant.

## Introduction

The Ubiquitin-Proteasome System (UPS) is considered to be a major pathway of protein-specific degradation and plays an important role in the signal pathway of regulating environmental stresses in the post-translation stage of protein (Serrano et al., 2018; Stone, 2019; Sharma and Taganna, 2020). The system involves the coordinated catalytic activities of three types of enzymes, namely a large number of ubiquitin ligases (E3), together with a few ubiquitin activating enzymes (E1) and ubiquitin-conjugating enzymes (E2) (Yan et al., 2003; March and Farrona, 2018). In the process of ubiquitination, when ATP supplies energy, E1 activates the ubiquitin molecules and transmits them to E2, and E3 connects the ubiquitin binding E2 to the target protein, and finally achieves ubiquitination of the target protein (Finley and Chau, 1991; Pickart, 2001; Trenner et al., 2022). Ubiquitin ligases are crucial in the ubiquitin pathway as they specifically recognize the target proteins for ubiquitination, and it is also the most abundant enzyme in terms of quantity. Studies have shown that the ubiquitin ligases are classified into different families according to their structure, function, and substrate specificity (Kim et al., 2021a), but functional domains of four families (HECT, RING, U-box, and cullin) are common. U-box protein contains a 70-amino acid U-box domain, a single protein widely distributed in yeast, plants, and animals (Shu and Yang, 2017). The first plant elucidated U-box protein (PUB) family was in *Arabidopsis*, which contains 64 members (Azevedo et al., 2001; Trujillo, 2018). Subsequent studies have successively identified 82 members in wild emmer wheat (Yang et al., 2021a), 70 in *Salvia miltiorrhiza* (Pan et al., 2022), 77 in rice (Kim et al., 2021b), 121 in *Phyllostachys edulis* (Zhou et al., 2021), 67 in barley (Ryu et al., 2019) and 62 in tomato (Sharma and Taganna, 2020).

Numerous studies have shown that U-box proteins are involved in the regulation of plant hormone signal transduction, abiotic and biotic stress responses (Zeng et al., 2008). For example, *AtU-box18* and *AtU-box19* were found to coordinately function as regulatory components in development and stress response in *Arabidopsis* (Bergler and Hoth, 2011). Furthermore, *NtACRE276* was confirmed to have the E3 ligase activity and involved in cell death and defense signaling, and its ortholog in *Arabidopsis* (*AtU-box17*) and canola (*BnARC1*) showed similar biological function (Yang et al., 2006). In wheat, *TaPUB1* plays a key role in regulating the antioxidant capacity of diploid wheat under drought stress (Zhang et al., 2017), enhancing its resistance to powdery mildew fungi by controlling U-box proteins of CMPG1-V (Zhu et al., 2015). Similarly, in tomato, *SIU-box13* and *SIU-box40* were found to confer resistance against tomato yellow leaf curl virus (Sharma and Taganna, 2020).

Tobacco and eggplant are important crops. Extensive studies have demonstrated that *U-box* genes play vital roles in regulating diverse developmental processes and stress signaling in plants (Azevedo et al., 2001). Current research on members of the *U-box* gene family in tobacco and eggplant is limited, and their exact function is still unknown. Therefore, it is of great significance to systematically analyze the *U-box* gene family in tobacco and eggplant. The purpose of this study was to comprehensively analyze the *U-box* gene family by integrating transcriptome data of tobacco and eggplant and study the expression patterns of *U-box* gene family members under pathogen *Ralstonia solanacearum* L. *(Ras)* infection conditions. The results of this study lay an important foundation for further analysis of the function and trait improvement of the *U-box* gene family in tobacco and eggplant.

## Materials and methods

### Genome identification of *U-box* gene family members in two Solanaceae species

The genome sequence and annotation data of Solanaceae species, including tomato (ITAG2.4), eggplant (SME-HQ) and tobacco (Nitab-v4.5) were downloaded from the Sol Genomics Network (https://solgenomics.net/) (Fernandez-Pozo et al., 2015; Edwards et al., 2017). The local protein database of tobacco and eggplant was constructed by command ‘makeblastdb’ of the local BLAST tool (BLAST+ 2.13.0) and a total of 62 known tomato U-box protein sequences were used as seed sequences to align with the tobacco and eggplant protein sequences by BLASTP program (Sharma and Taganna, 2020) U-box domain (PF04564) was obtained from the Pfam database (version 37.0) (http://pfam.xfam.org/) (Mistry et al., 2021).Sequences with the similarity ≥30% and E≤ 1e^−10^ were considered as the candidate proteins. Subsequently, the candidate protein sequences were further analyzed for the presence of a U-box structure domain (PF04564) using the CDD program of NCBI (https://www.ncbi.nlm.nih.gov/cdd/) and SMART tool (http://smart.embl-heidelberg.de/). The candidate protein containing the U-box conserved domain was confirmed as the final U-box protein. These *U-box* genes of tobacco and eggplant were renamed as *NtU-boxs* and *SmU-boxs*, respectively. The physicochemical properties of the tobacco and eggplant U-box proteins were predicted and analyzed using the ExPASy software (https://www.expasy.org) (Wilkins et al., 1999) and the subcellular location of each U-box protein analysis was based on the Cell PLoc 2.0 (Chou and Shen, 2008). The gene structure, conserved motif, phylogenetic tree, chromosomal localization, and synteny were analyzed, and corresponding flow chart was provided in **Supplementary Figure S1**.

### Gene structure and conserved motif analysis

The GFF format file of gene structure for tobacco and eggplant was obtained from the Solanaceae genome database (https://solgenomics.net/) (Fernandez-Pozo et al., 2015; Edwards et al., 2017).The intron-exon gene structures of *NtU-box* and *SmU-box* genes were displayed using TBtools (version 2.097) (Chen et al., 2020) based on the gff3 files of the tobacco and eggplant genome. The conserved motifs of NtU-box and SmU-box proteins were analyzed using the online program MEME (https://meme-suite.org/meme/tools/meme) (Ma et al., 2014; Bailey et al., 2015) and the parameters were as follows: the number of motifs was set to 20, and the width range of motifs was set to be 5-200 amino acids respectively. Motif annotation was identified using the Pfam online tool (http://pfam-legacy.xfam.org/). *U-box* genes were submitted to the PlantCARE online program (Lescot et al., 2002) (http://bioinformatics.psb.ugent.be/webtools/plantcare/html/) for *cis*-acting elements prediction.

### Multiple sequence alignment and phylogenetic analysis

To explore the evolutionary relationship of the *U-box* gene family in plants, the full protein sequences of U-box from tobacco, eggplant, pepper, potato, tomato, and *Arabidopsis* were analyzed. The Clustal X software (Thompson et al., 1997) was used to perform multiple sequence alignment. The phylogenetic tree was constructed by the MEGA-11 (Tamura et al., 2021) tool using the maximum likelihood method (ML) with a bootstrap of 1000 replications. The ML is a significant statistical method for parameter estimation. The ITOL (version 6.0) tool (https://itol.embl.de/) was used to edit the phylogenetic tree of NtU-box proteins and SmU-box proteins.

### Chromosomal localization and synteny analysis

Based on the annotation information and the full genome protein sequences of tobacco (Nitab-v4.5), eggplant (SME-HQ), tomato (ITAG2.4) and *Arabidopsis* (TAIR10), the MCScanX software (Wang et al., 2012) with default parameters was employed to analyze the possible segmental duplication, tandem duplication events, intra-genomic syntenic and inter-genomic collinearity blocks (Edwards et al., 2017) and the TBtools software (version 2.097) (Chen et al., 2020) was used for visualization.

### Expression analysis of *NtU-box* and *SmU-box* genes

To investigate the expression patterns *U-box* genes across different tissues and under various abiotic stresses, we analyzed the FPKM (Fragments Per Kilobase of transcript per Million mapped reads) values of *NtU-box* genes in different tissues (GSE233199) (Mo et al., 2023) under drought conditions (GSE214048) (Hu et al., 2022), as well as *SmU-box* genes in different tissues (PRJNA328564) (Barchi et al., 2019) and under high temperature conditions (Liu et al., 2023), which were download from NCBI(https://www.ncbi.nlm.nih.gov/geo/). A map was generated using the heatmap function of the R gplots package (Walter et al., 2015).

The Hongda variety of tobacco and Yanzhi variety of eggplant were cultivated using conventional cultivation methods. The seedlings were managed until the 3-5 leaf stage. A total of 100 tobacco seedlings and 100 eggplant seedlings were selected and inoculated with a highly efficient strain of *Ralstonia solanacearum* L. (*Ras*) that had been isolated and maintained by our laboratory (Gao et al., 2019). These plants were cultured in a greenhouse with high-humidity and high-temperature (approximately 80% humidity, 28~30°C, 14 h light exposure; 10 h dark environment). The seedlings of tobacco and eggplant were collected at 0 h, 12 h, 24 h, 48 h and 96 h after inoculation, with each biological sample consisting of 5 plants and a total of 3 replicates. For sampling, seedlings were uprooted and their roots were quickly washed with sterile water to remove any attached soil and pathogens. The cDNA synthesis was carried out using the SMART Kit (Takara). To evaluate the expression levels of the *NtU-box* and *SmU-box* genes, real-time quantitative PCR (qRT-PCR) was conducted using SYBR Green qPCR Premix (Universal), and the relative expression levels were calculated using the 2^−ΔΔt^ method (Livak and Schmittgen, 2001). Three technical replicates were performed for each sample. The actin genes of both tobacco and eggplant were used as the internal reference gene, and the primers of *NtU-box* and *SmU-box* genes (**Supplementary Table S1**) were designed using primer3 software (https://bioinfo.ut.ee/primer3-0.4.0/).

## Results

### Characterization and distribution of *U-box* genes in tobacco and eggplant genomes

In this study, a total of 116 *U-box* genes were identified in tobacco, while 56 *U-box* genes were in eggplant. These genes were renamed from *NtU-box1* to *NtU-box116* and *SmU-box1* to *SmU-box56*, respectively. To characterize these *NtU-box* and *SmU-box* genes, gene ID and protein molecular weight (MW), theoretical isoelectric point (pI), subcellular localization, number of exon, and CDS sequences were analyzed (**Supplementary Table S2**). In tobacco, *NtU-box* genes contained 1 to 19 exons, and the relative molecular weight of their corresponding proteins varied greatly from 20890.72 Da (*NtU-box112*) to 194534.46 Da (*NtU-box31*), and the theoretical isoelectric point ranged from 5.12 (*NtU-box47*) to 9.47 (*NtU-box110*). In eggplant, *SmU-box* genes contained 1 to 18 exons. The predicted molecular weight ranged from 31784.29 Da *(SmU-box53)* to 166473.85 Da *(SmU-box43)*, while the theoretical isoelectric point varied from 4.92 *(SmU-box51)* to 9.23 *(SmU-box9)*. Subcellular localization analysis of NtU-box and SmU-box proteins showed that these proteins were mainly present in the nucleus. Among the 116 NtU-box proteins, 5 were located on the cytoplasm, 4 on the cell membrane, and 1 on the chloroplast, while other NtU-box proteins were located on the nucleus. Among the 56 SmU-box proteins, 3 were located in the cytoplasm, 1 was located in the chloroplast, and the other SmU-box proteins were located in the nucleus (**Supplementary Table S2**).

### Chromosome localization and collinearity analysis of *NtU-box* and *SmU-box* genes

**Figure 1:**
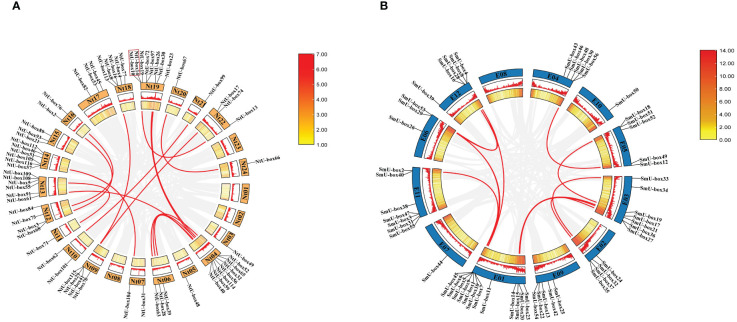
Gene distribution and duplication **(A)** *NtU-box* genes on the 24 chromosomes in tobacco **(B)** *SmU-box* genes on the 12 chromosomes in eggplant. Tandem-duplicated gene pairs of Nt19 are marked with red box, and segmental duplication genes are connected by red line.

The analysis of chromosomal localization showed that some *NtU-box* genes could not acquire the precise location information due to the incomplete sequencing of the tobacco genome. Among the 116 *NtU-box* genes, a total of 64 genes were unevenly distributed on 24 chromosomes of tobacco, while the remaining 52 *NtU-box* genes were mapped to unassigned scaffolds (**Figure 1A**). Notably, chromosome 19 contained the largest number of *NtU-boxs* (8 genes), followed by chromosome 04 with 7 *NtU-box* genes, and chromosomes 13 and 14 with 6 *NtU-box* genes each. In contrast, chromosomes 03, 05, 11, 20, 21, and 24 each contained only one *NtU-box* gene, while chromosomes 01, 02, 08 and 23 had no *NtU-box* genes detected (**Figure 1A**). In addition, 1 pair of tandem duplication genes on chromosome 19 (*NtU-box14/10*) and 15 pairs of segmental duplication genes were identified in the tobacco *NtU-box* gene family (**Figure 1A**, **Supplementary Table S3**). The result of chromosomal location analysis revealed that 55 out of 56 *SmU-box* genes were unevenly distributed among the 12 chromosomes of eggplant, with only 1 *SmU-box* gene located on an unattributed scaffold. Chromosome 01 contained the largest number of *SmU-boxs* (12 genes), followed by chromosomes 03 and 11 with 7 *SmU-box* genes, and 5 *SmU-box* genes for chromosomes 04, 05, 09 and 12. However, chromosomes 02, 06, 07, and 10 contained 4, 3, 1, and 1 *SmU-box* genes, respectively, while none of the *SmU-box* genes were detected on chromosome 08 (**Figure 1B**). In the eggplant *SmU-box* gene family, 10 pairs of fragment duplication genes were identified, but no tandem repeats were identified (**Figure 1B**; **Supplementary Table S3**).

**Figure 2:**
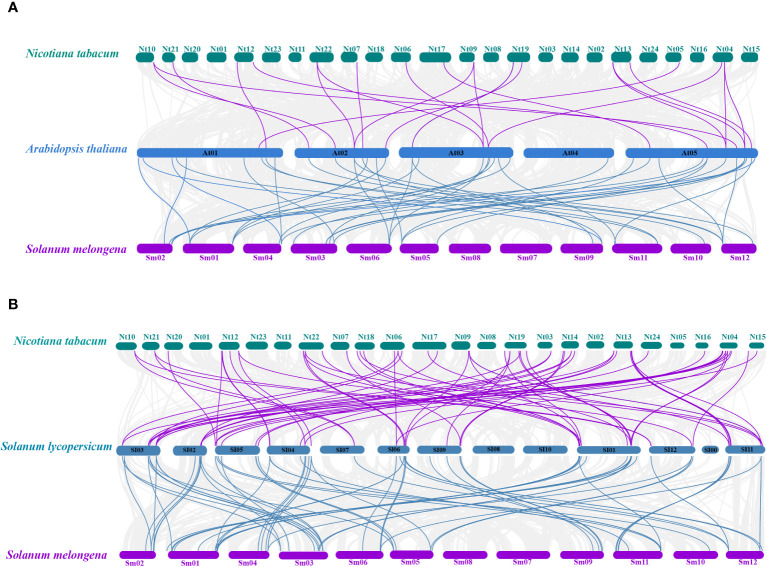
Collinearity analyses of *U-box* genes among tobacco, eggplant tomato and *Arabidopsis*. **(A)** Collinearity analyses among *Arabidopsis thaliana*, *Nicotiana tabacum* and *Solanum melongena*. **(B)** Collinearity analyses among *Solanum Lycopersicon*, *Nicotiana tabacum* and *Solanum melongena*. The gray line represents the co-collinearity of all genes among the three species, and the purple and blue line represents the collinearity among members of the *U-box* gene family.

A total of 22 orthologous genes were identified between tobacco and *Arabidopsis* based on the interspecies syntenic analysis, while there were 65 syntenic counterparts between tobacco and tomato (**Figures 2A, B**). A total of 68 orthologous genes were identified between eggplant and *Arabidopsis* based on the interspecies syntenic analysis, while there are 41 syntenic counterparts between eggplant and tomato (**Figures 2A, B**). The genomic regions around *NtU-box41/61/62/74*, *SmU-box9/15/29/39/40* showed strong syntenic relationships with their counterparts in both *Arabidopsis* and tomato (**Supplementary Table S4**). Notably, good collinearity was detected among the *U-box* genes of four distinct species, even after undergoing speciation and long-term evolution, and the result suggested that these genes might have originated before Solanaceae species diversification and retained conserved functional roles.

### Phylogenetic and gene structure analysis of *NtU-box* and *SmU-box* genes

**Figure 3:**
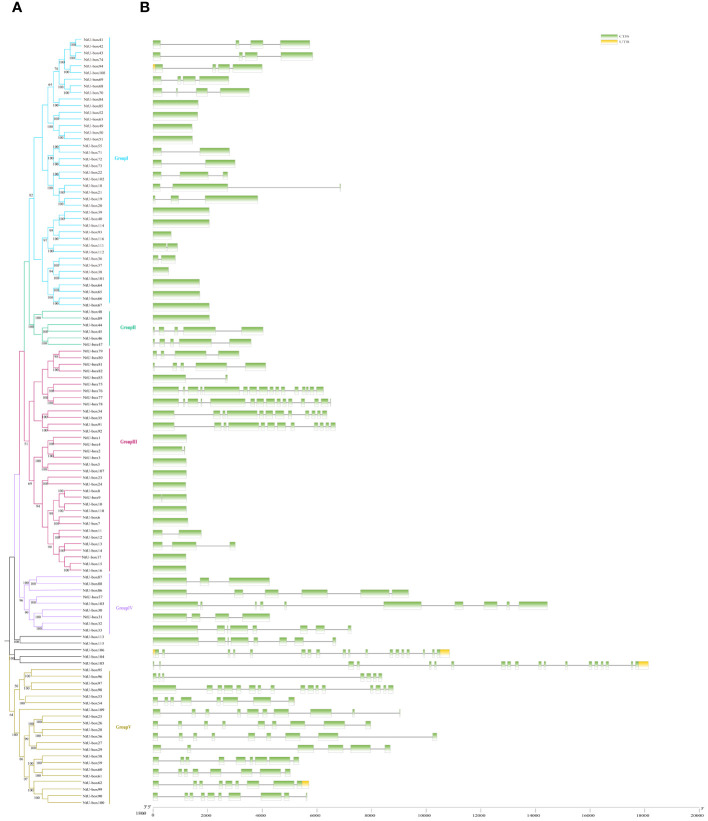
Gene structure and evolution of U-box family in tobacco **(A)** Phylogenetic relationships of *NtU-boxs*. Different subgroups were marked with different colors. **(B)** Intron-exon structure of *NtU-boxs*. UTR and CDS are represented by different colors. The scale bar of bottom demonstrates the length of exons and introns.

**Figure 4:**
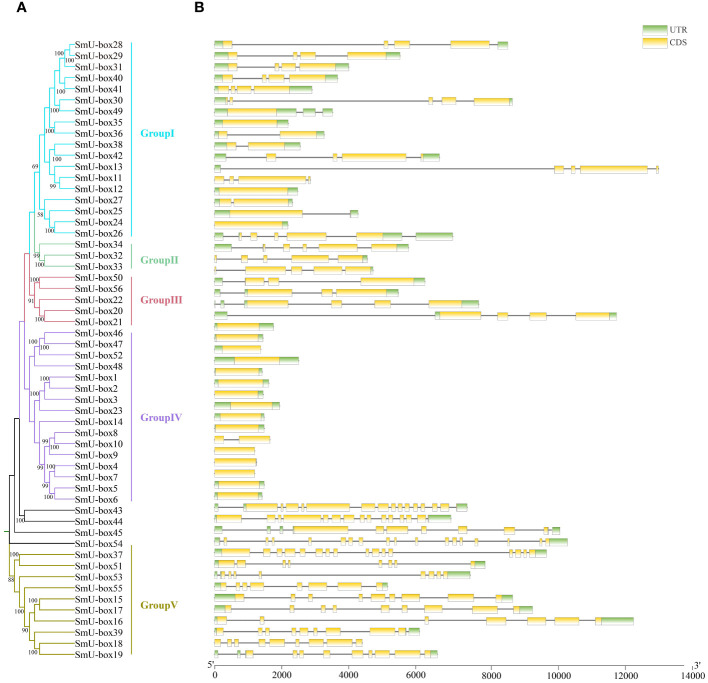
Gene structure and evolution of U-box family in eggplant. **(A)** Phylogenetic relationships of *SmU-boxs*. Different subgroups were marked with different colors. **(B)** Intron-exon structure of *SmU-boxs*. UTR and CDS are represented by different colors. The scale bar of bottom demonstrates the length of exons and introns.

To investigate the evolutionary relationship between *NtU-box* and *SmU-box* genes, a phylogenetic tree was constructed (**Figures 3A**, **4A**). In tobacco, *NtU-box* genes were divided into 5 subgroups (I ~ V), with the largest members (41 members) in subgroup I. The subgroup represented more than 35.3% of the total *NtU-box* members. In contrast, subgroups II, IV, and V had only 6, 9 and 21 members, respectively. In eggplant, the largest members of eggplant (18 members) found in the subgroup I and this subgroup represented more than 32.1% of the total *SmU-box* members. In contrast, subgroups II, III, and V had only contained 3, 5 and 10 members, respectively. Gene structure of *NtU-boxs* were found that the number of exons varied from 1 (*NtU-box1*) to 19 (*NtU-box105*) (**Figure 3B**).

The number of exons *SmU-box* gene ranging from 1 to 18. Among 116 *U-box* genes in tobacco, *NtU-box105* and *NtU-box106* contained the greatest number of exons (19), while 40 *NtU-box* genes (34.5%) only contained one exon (**Figure 3B**). Among 56 *U-box* genes in eggplant, 21 *SmU-box* genes (37.5%) contained one exon (**Figure 4B**). In addition, the *U-box* genes with similar gene structures were clustered into the same sub-clade. For example, most tobacco members of Group II only housed five exons. This result indicated that the members of the same groups exhibited similar gene structures.

### Domain and motif analysis of the NtU-box and SmU-box proteins

**Figure 5:**
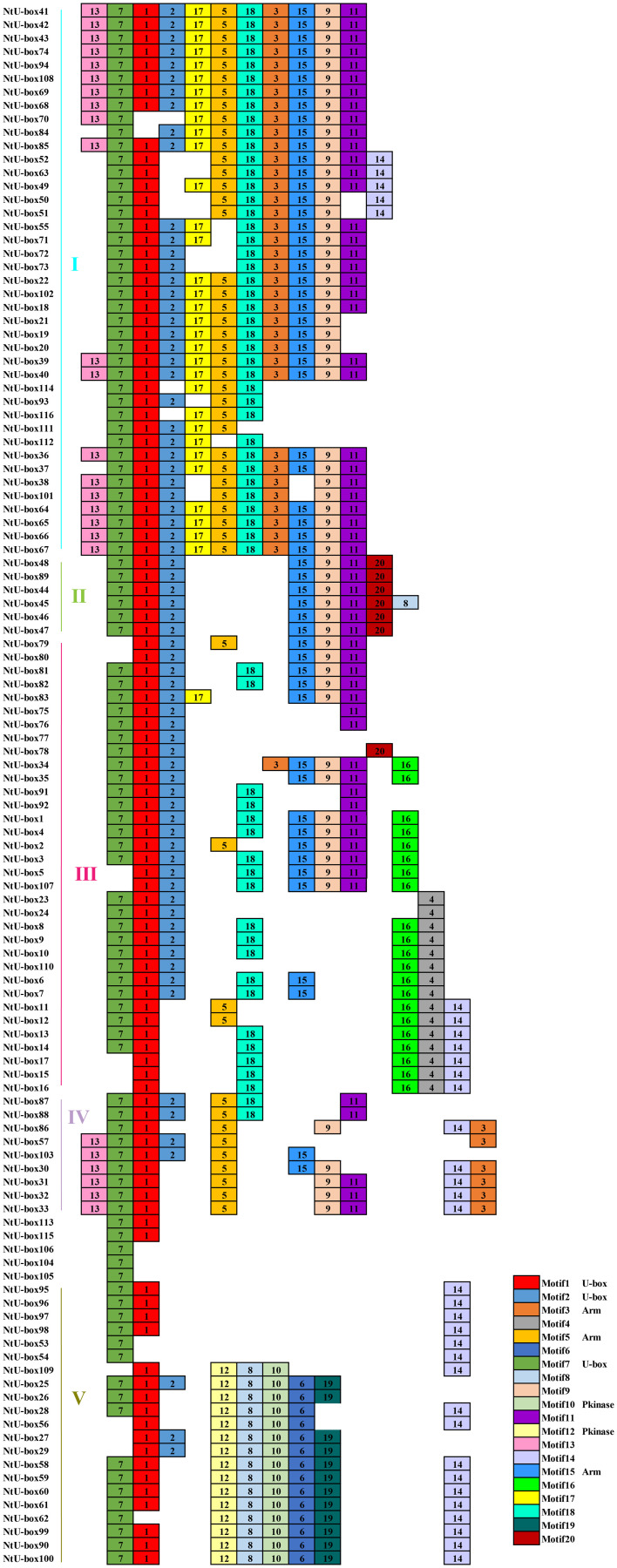
Conserved motifs for U-box proteins in tobacco. Different motifs are showed with different colored boxes and numbers (1-20).

**Figure 6:**
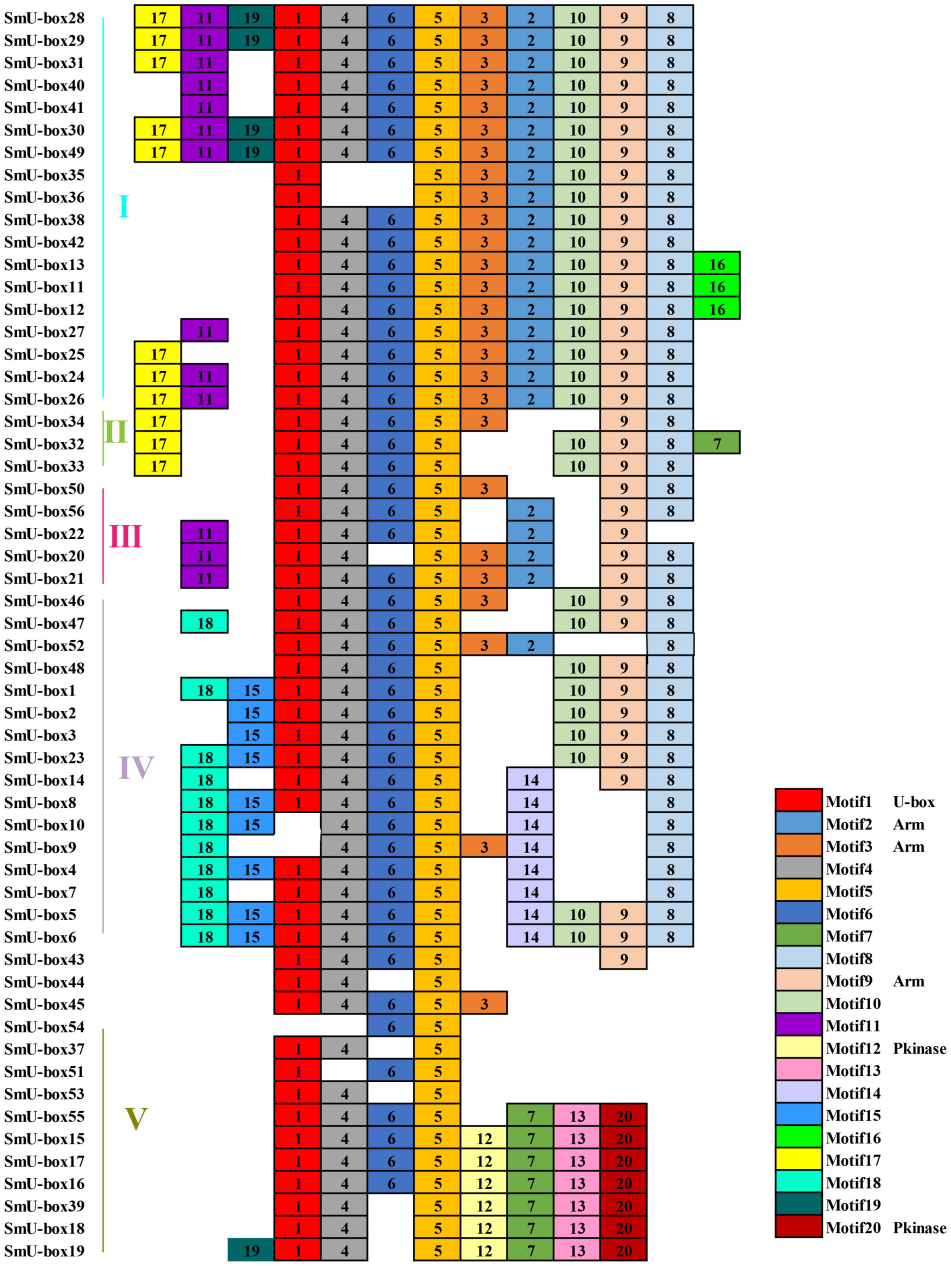
Conserved motifs for U-box proteins in eggplant. Different motifs are showed with different colored boxes and numbers (1-20).

A total of 20 conserved motifs have been identified in the 116 *NtU-box* and 56 *SmU-box* genes. The lengths and conserved sequences of each motif are listed in **Supplementary Table S5**. Among them, Motif 7 and Motif 1 were prevalent across most genes in all five groups of tobacco NtU-box proteins (**Figure 5**). Similarly, in eggplant SmU-box proteins Motif 1, Motif 4, Motif 6, and Motif 5 were found in most genes of the five groups, indicating their high conservation in U-box proteins (**Figure 6**). The protein sequences of the 20 motifs were uploaded to the CDD program for further domain analysis. Motif 1, Motif 2, and Motif 7 were annotated as components of the conserved U-box domain sequences, essential for maintaining the structural integrity of the U-box and facilitating ubiquitin linkage activity. Additionally, Motif 3 was annotated as part of the ARM conserved domain, which was the most common type in the U-box family. Moreover, genes in the same group on the phylogenetic tree exhibited similar conserved motifs. For example, all *NtU-box* genes in Group II had the same 7 motifs (Motifs 1, 2, 7, 9, 11, 15, and 20), suggesting potential functional similarities.

### Phylogenetic analysis and functional prediction of the *U-box* gene family in Solananceae species

**Figure 7:**
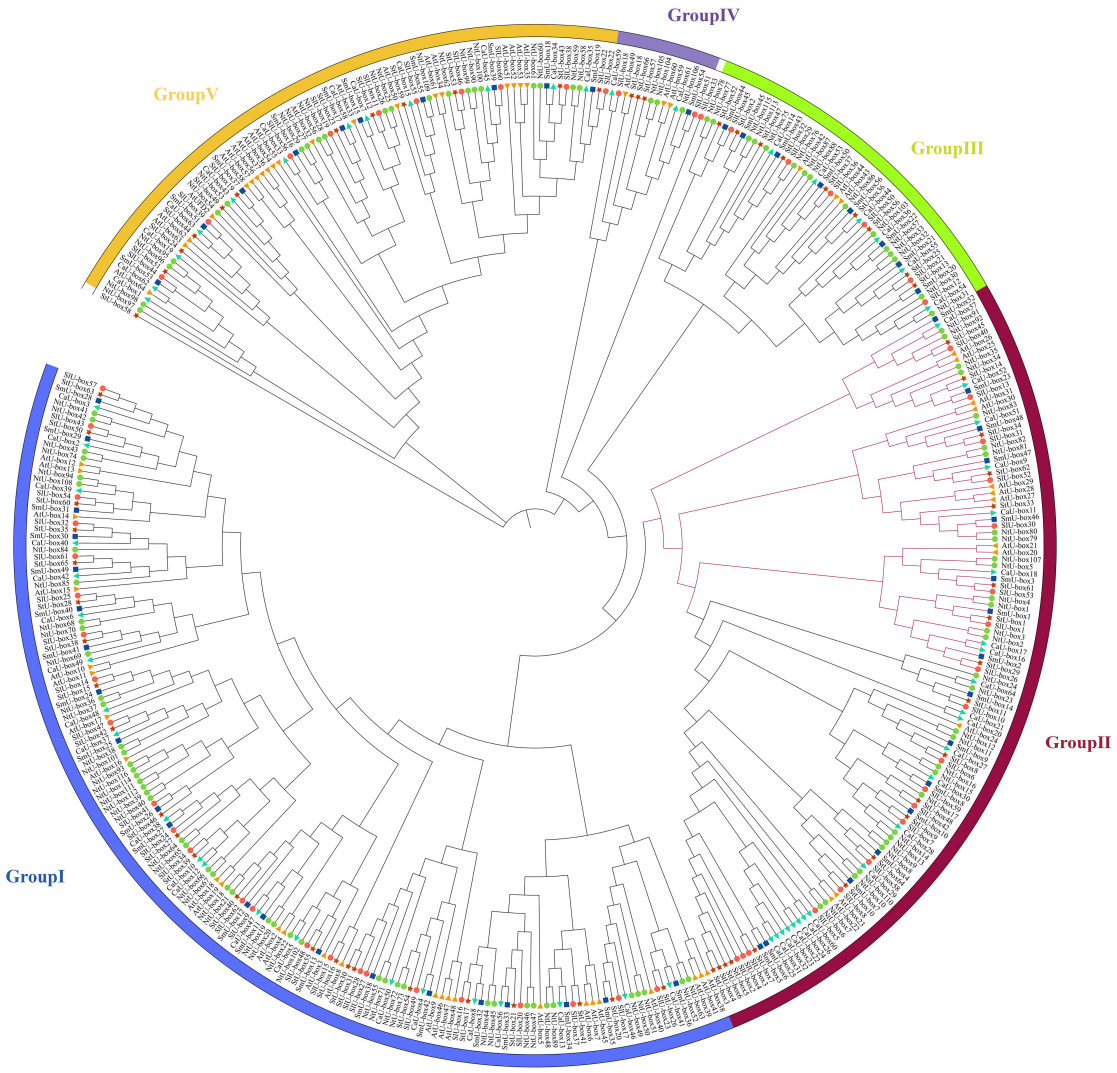
Phylogenetic relationship of 116 NtU-box and 56 SmU-box proteins, along with another 256 published U-box proteins. The phylogenetic relationships were generated by using MEGA-11 using the Maximum Likelihood (ML) method (1000 bootstrap replicates), and visualized with ITOL software. U-box proteins were classified into five distinct groups, as indicated by the different colors.

To explore the evolution of the *U-box* gene family in plant, 428 *U-box* gene members from 6 species were selected to construct a phylogenetic tree (**Figure 7**), including tobacco (116), eggplant (56), potato (66), tomato (62), *Arabidopsis thaliana* (64), and pepper (64) (**Supplementary Table S6**). Based on previous study, 428 *U-box* genes were divided into 5 different subfamilies (Wang et al., 2021). Based on the phylogenetic tree, a total of 138 sister pairs of homologous proteins were identified, including 71 pairs of orthologous genes and 67 pairs of paralogous genes (**Supplementary Table S7**). Specifically, there were 41 paralogous pairs from tobacco, 17 pairs from *Arabidopsis thaliana*, 6 pairs from pepper, 2 pairs from potato, and 1 pair from eggplant.

Similarity in gene expression patterns implies similar functions, especially for homologous genes (Yang et al., 2021b). Based on published transcriptomic data, the expression patterns of *U-box* genes in major tissues were compared in tobacco (116), eggplant (56), and *Arabidopsis thaliana* (64). These *NtU-box* and *SmU-box* genes were classified into 7 categories according to their normalized expression levels (**Supplementary Figure S2A**). Among them, the *U-box* genes of group I specifically expressed in leaf with a high level; the *U-box* genes of group II expressed specifically high in all tissue; *U-box* genes in Cluster III mostly expressed in stem; *U-box* in group IV and V highly expressed in roots. Based on the tissue expression clustering characteristics, phylogenetic relationship of multispecies *U-box* genes were predicted (**Supplementary Table S8**).

In general, the functions of some *NtU-box* and *SmU-box* genes were mainly divided into the following four categories: 1. Play a role in formation of organs and regulator of flowering time. 2. Play a role in regulating root development, pollen tapetum development and ROS induced chloroplast degradation. 3. Play combinatory roles in response to drought stress. 4. Encodes a U-box domain-containing E3 ubiquitin ligase with central Ser/Thr protein kinase domain, and its expression is responsive to both phosphate (Pi) and phosphite (Phi) in both roots and shoots (**Supplementary Table S8**). Only those *NtU-box* and *SmU-box* genes that were homologous to the reported *AtU-box* genes, had highly similar expression patterns, and shared similar functions. Fox example, *AtPUB18* and *AtPUB19* function as regulators in the drought stress response (Liu et al., 2011) and their homologous genes *NtU-box65/66/67* genes and *SmU-box27* genes, which had similar expression patterns, were predicted to have similar functions (**Supplementary Figure S2B**). Transcriptome data showed that *NtU-box65/66/67* genes and *SmU-box27* genes showed a positive regulatory expression pattern under heat stress (**Supplementary Table S9**). *AtPUB14* plays a role in organ formation and flowering time regulation (Feke et al., 2020), and its homologous gene *SmU-box28/29/31* has a similar expression pattern in eggplant (**Supplementary Figure S2C**). Transcriptome data showed that *SmU-box28/29/31* has a high expression level in eggplant flowers, which is expected to have similar functions with *AtPUB14*.

### *Cis*-regulatory elements analysis and tissue expression patterns of *U-box* genes

**Figure 8:**
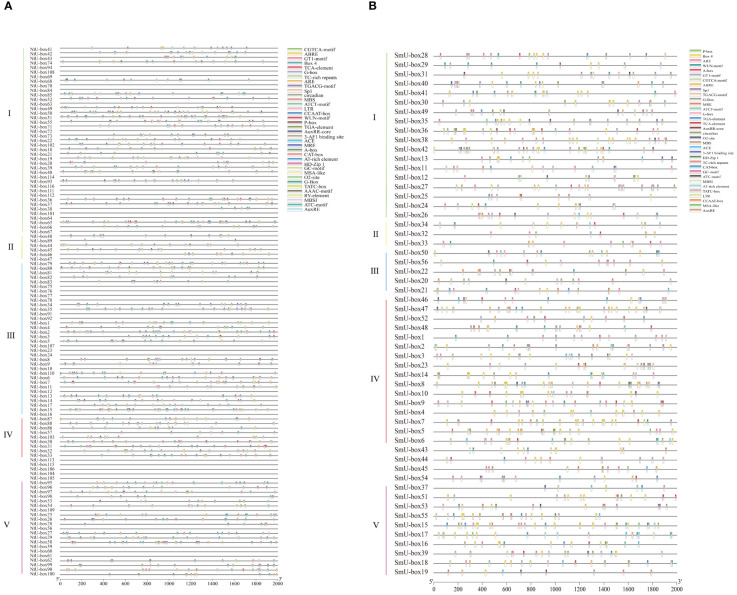
**(A)** Predicted *cis*-elements in *NtU-box* promoters. **(B)** Predicted *cis*-elements in *SmU-box* promoters. Different shapes and colors represent the different types of *cis*-elements. Annotations of *cis*-elements were listed in **Supplementary Table S10**.

To investigate the potential function of *U-box* genes during plant development and upon exposure to various stresses, the *cis*-elements within the promoter regions of *U-box* genes were analyzed. A total of 36 *cis*-regulatory genes were identified in the promoter region of *NtU-box* genes, while 34 *cis*-regulatory genes were identified in the promoter region of *SmU-box* genes (**Figure 8**, **Supplementary Table S10**). These *cis*-regulatory genes can be divided into four categories, specifically cell development, plant hormones, environmental stress, and photoresponse elements (**Supplementary Table S10**). The 5 *cis*-acting elements related to cell development include CAT-box, MSA-like, CCAAT-box, MBSI, and HD-Zip 1. For phytohormone-responsive elements, the *cis*-acting elements include TGACG-motif, ABRE, P-box, TGA-element, TCA-element, AuxRR-core, TATC-box, GARE-motif, AuxRE, A-box, and O2-site. Additionally, ten light responsive elements were identified, including GT1-motif, G-Box, Box4, MRE, ATC-motif, Sp1, ATCT-motif, ACE, 3-AF1 binding site, and AAAC-motif. The expression of these genes might be regulated by phytohormones, various light-responsiveness *cis*-elements, defense signaling transduction, and abiotic stresses during the growth of tobacco and eggplant.

**Figure 9:**
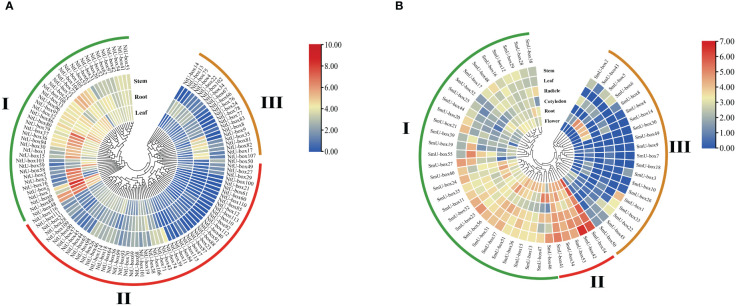
The expression patterns of tobacco and eggplant *U-box* genes in different tissues. **(A)** The expression patterns of *NtU-box* genes **(B)** The expression patterns of *SmU-box* genes. FPKM values for *U-box* genes were transformed by log2 (n+1).

Transcriptome analysis revealed diverse expression patterns of *NtU-box* and *SmU-box* genes in different tissues, which were clustered into three groups (**Figure 9**) (Yang et al., 2022). In tobacco (**Figure 9A**), group I comprised 41 *NtU-box* genes, with the majority showing high levels of expression in vegetative tissue, particularly in root. Conversely, *NtU-box* genes in groups II and III exhibited low levels of expression. A total of 5 *SmU-box* genes (*SmU-box* 54/42/53/34/41) in group II showed high levels of expression in all investigated tissues, including stem, leaf, radicle, cotyledons, root, and flower (**Figure 9B**), and 31*SmU-box* genes in group I exhibited moderate levels of expression. Conversely, *SmU-box* genes in group III showed nearly negligible expression, exhibited lower expression levels.

### The expression profiles of tobacco and eggplant *U-box* genes under various abiotic and biotic stresses

**Figure 10:**
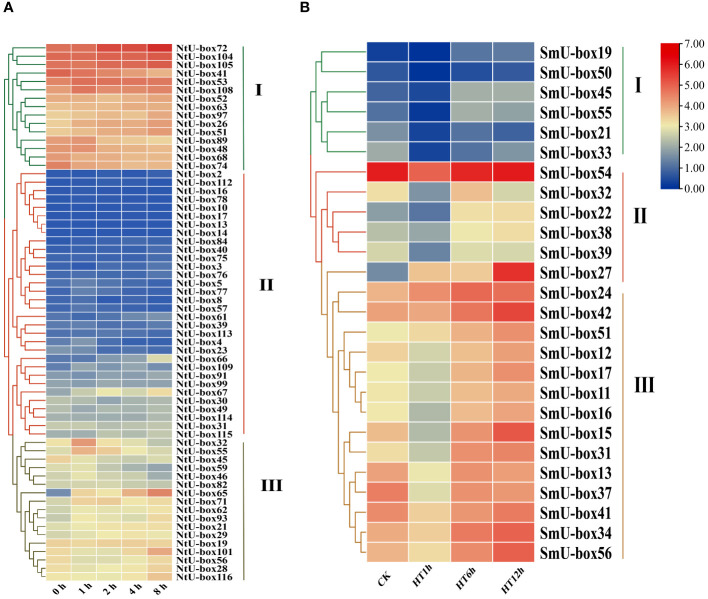
The expression profiles of tobacco and eggplant *U-box* genes under abiotic stresses. **(A)** The expression patterns of *NtU-box* genes under drought stress. **(B)** The expression patterns of *SmU-box* genes under high temperature. FPKM values for *U-box* genes were transformed by log2 (n+1).

To determine the expression profiles of *U-box* family genes under abiotic stress, the FPKM data of tobacco *NtU-box* genes under drought stress (Hu et al., 2022) and eggplant *SmU-box* genes under high temperature (Liu et al., 2023) were downloaded. The expression profiles of 62 *NtU-box* genes and 26 *SmU-box* genes were analyzed (**Supplementary Table S9**). The results revealed distinct expression patterns among the *NtU-box* and *SmU-box* gene members under various stress conditions (**Figure 10**). Specifically, the 62 *NtU-box* genes and 56 *SmU-box* genes were each classified into three groups (I ~ III). In tobacco, a total of 15 *NtU-box* genes were included in group I, and the high expression levels of these genes in the five drought stress stages mean that these genes can play an important role in the drought stress process, while the 32 *NtU-box* genes clustered in group II showed low or no expression in the whole drought stress process. In eggplant, a total of 14 *SmU-box* genes were included in group III, and the expression profiles of these genes exhibited a pattern of initial decrease followed by an increase across the three high-temperature stress stages, indicating their potential significance in responding to high-temperature stress. Conversely, the 6 *SmU-box* genes in group I showed either minimal or no expression throughout the high-temperature stress process. It is worth noting that the expression levels of *NtU-box59* and *NtU-box46* genes gradually decreased with the increase of drought time. In terms of high-temperature stress (**Figure 10B**), the expression level of the *SmU-box27* gene in group II gradually increased with the increase of high-temperature time, while the majority of *SmU-box* genes (*SmU-box54/32/22/38/39*) reached the peak expression level at the 12 h of high-temperature stress. These results indicated the functional diversity of *U-box* members among different species.

**Figure 11:**
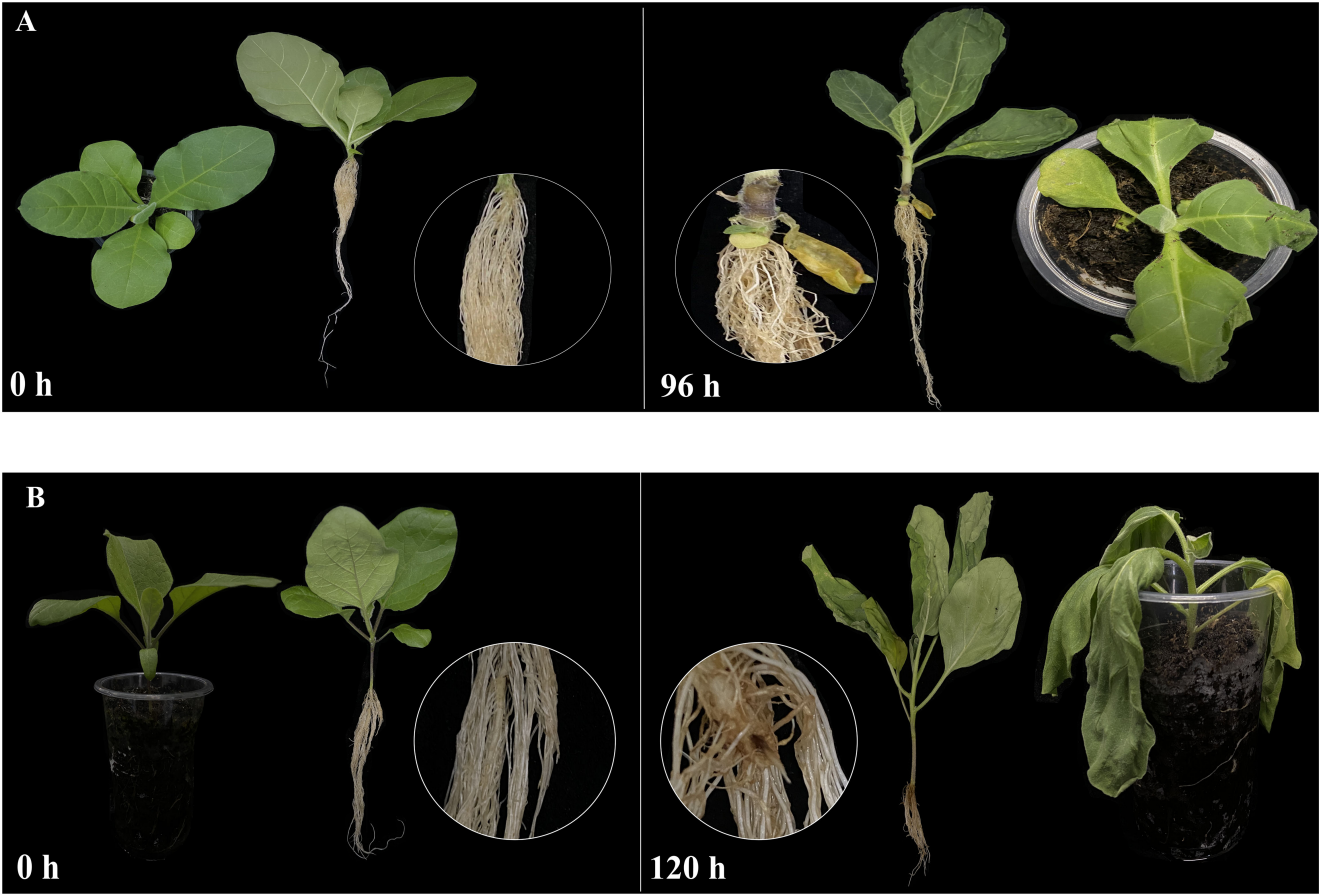
Disease symptoms of tobacco and eggplant seedlings. **(A)** Tobacco seedlings at 0 h and 96 h post-inoculation with *Ras*. **(B)** Eggplant seedlings at 0 h and 120 h post inoculation with *Ras*. The basal parts of stems were magnified and shown in the circles.

**Figure 12:**
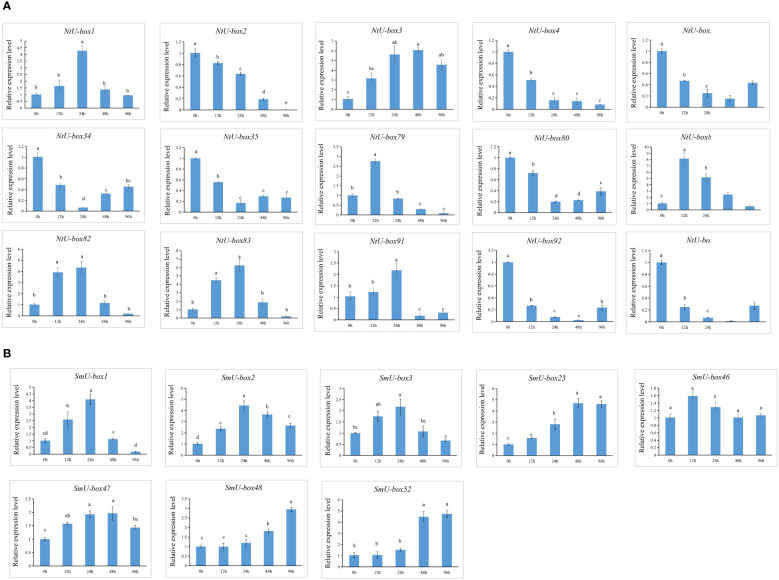
Expression profiles of *U-box* genes in response to *Ras* infection. **(A)** Relative expression level of 15 *NtU-boxs* in response to *Ras* infection. **(B)** Relative expression level of 8 *SmU-boxs* in response to *Ras* infection. Error bars are standard deviations of three biological replicates. Diverse letters on top of the bars indicate significant differences at *P*<0.05.

No significant changes were observed in the seedlings of both tobacco and eggplant at the initial stage after being infected by *Ralstonia solanacearum* L. (*Ras*). In tobacco, the primary symptoms induced by *Ras* infection manifested in the seedling at 96 h (**Figure 11A**). During this stage, the seedling exhibiting leaf wilting and stem necrosis, with the roots turning yellow and exhibiting necrosis. In contrast, symptoms in the eggplant appeared later than those in tobacco, with notable symptoms appeared at 120 h (**Figure 11B**). The leaves appeared withered, and the basal part of the stem showed severe necrosis and turned black. It was reported that *SIU-box13* and *SIU-box40* in tomato, play a crucial role in the regulation of pathogen invasion (Sharma and Taganna, 2020). To further explore the potential functions of the *U-box* genes in tobacco and eggplant, a total of 15 *NtU-box* and 8 *SmU-box* genes that clustered with *SIU-box13* and *SIU-box40* in II subgroups of the phylogenetic tree (**Figure 7**) were selected for qRT-PCR analysis under *Ras* infection. The qRT-PCR analysis found that the majority of the selected genes showed significant response to *Ras* infection. In tobacco (**Figure 12A**), the expression levels of 7 *NtU-box* genes (*NtU-box1/3/79/81/82/83/91*) exhibited a trend of initial increased followed by decreased with the extension of time after inoculation. Among these genes, the expression levels of 5 genes (*NtU-box3/79/81/82/83*) were significantly up-regulated at 12 h after inoculation, while 2 genes (*NtU-box2/4*) displayed significant down-regulated and 6 genes (*NtU-box5/34/35/80/92/107*) showed a pattern of decrease followed by a slight increase. In eggplant (**Figure 12B**), all the 8 selected genes displayed up-regulated expression in response to the infection compared to the initial stage (0 h), and 2 genes (*SmU-box1/2*) exhibited significant up-regulation exceeding a 4-fold increase at 24 h post-inoculation.

## Discussion

*U-box* gene family members are present in almost all eukaryotes. The advent of genome sequencing has facilitated comprehensive analyses of the *U-box* gene family in numerous species, such as *Arabidopsis thaliana* (64), rice (77), soybeans (125), and tomato (62) (Wiborg et al., 2008; Zeng et al., 2008; Wang et al., 2016; Sharma and Taganna, 2020). In this study, a total of 116 *U-box* genes were identified in tobacco, which were larger than those in *Arabidopsis thaliana* and rice, but less than in soybean. As an allotetraploid, the genome size of *Nicotiana tabacum* is 4.5Gb, while that of Arabidopsis, rice and soybean is 125Mb, 430Mb and 1.025Gb, respectively (Kaul S et al., 2000; Burr, 2002; Schnable et al., 2009; Schmutz et al., 2010). In this case, it seems that there is no direct correlation between the number of *U-box* genes and genome size in these plants. The number of exons observed between *NtU-box* genes varies significantly, of which 64 genes contain two or more exons, 13 genes have more than 10 exons, and 39 genes possess only one exon. Similarly, for *SmU-box* genes, differences are notable: 32 genes contain two or more exons and 4 genes have more than 10 exons, and however, 20 genes possess only one exon. This suggested that distinct RNA splicing processes that could modulate the proportion of isoforms (active and non-active), crucial for stress regulation (Tang et al., 2021). Many *SmU-box* genes are either intron-less or have two introns, consistent with reports in tomato (Sharma and Taganna, 2020). *U-box* genes with multiple introns may act as a mutational buffer, protecting coding sequences from deleterious mutations. The presence of the intron-less genes underscores structural integrity within the *U-box* gene family, suggesting irregular distribution of U-box protein in different species (Zeng et al., 2008). The phylogenetic analysis of the *U-box* gene family showed a great similarity among all the five classes due to the presence of the core U-box domain in all the members (**Figure 7**), suggesting a shared ancestor predating the divergence of these species. Moreover, a single tandem duplication gene pair and 15 segmental duplication gene pairs were detected in tobacco (**Figure 1A**), while 10 segmental duplication gene pairs were identified in eggplant (**Figure 1B**), highlighting segmental duplication events as the primary driver for the expansion of the *U-box* gene family in both tobacco and eggplant. In addition, a good collinearity was detected among the *U-box* genes of four distinct species, even after undergoing speciation and long-term evolution. Syntenic analysis revealed a higher number of orthologous gene pairs between tobacco and tomato compared to those between tobacco and *Arabidopsis* (**Figures 2A, B**). Conversely, the count of orthologous gene pairs between eggplant and *Arabidopsis* exceeded those between eggplant and tomato (**Figures 2A, B**). The results suggested that species with close evolutionary relationships tend to exhibit greater similarity, higher homology, and increased conservation of the *U-box* genes.

In general, the evolution of gene family is predominantly determined by the organization of gene structures, whereas within a gene family, members of the same subfamily typically exhibit high conservation in both structure and function, reflecting their evolutionary relatedness. In this study, a total of 20 conserved motifs were identified in the *U-box* gene families of both tobacco and eggplant. Interestingly, despite belonging to the same or different subfamilies, *U-box* members exhibited variations in motif types and quantities. However, the differences in the same subfamily were notably smaller, indicating a higher level of conservation in motif composition within closely related members. These observations highlight the complexity of the tobacco and eggplant genomes and the differentiation and diversity of the function within the *U-box* gene family.

*U-box* genes play important roles in plant responses to both abiotic and biotic stresses (Adler et al., 2017; Sharma and Taganna, 2020; Tang et al., 2021). Functional analysis has revealed the molecular mechanisms involving U-box proteins involving U-box proteins in theses stress response (Cho et al., 2017). However, the role of the *U-box* gene family in tobacco and eggplant remains unclear. Stress refers to the phenomenon that plants are exposed to adverse conditions in their environment, causing their physiological processes to be negatively affected. Stress is usually divided into two categories: abiotic stress and biotic stress. Abiotic stress mainly refers to the unfavorable conditions caused by environmental factors, such as temperature, light, humidity, drought, salt and alkali. Biotic stresses include a range of biological factors that are harmful to plant survival and development, often stemming from infections and competition, including diseases, pests, and weeds (Atkinson and Urwin, 2012). These stress factors significantly affect crop growth and production. Therefore, mining excellent resistance genes in plants has become one of the main strategies to cope with various stress challenges. In this study, 116 *NtU-boxs* were identified in tobacco and 56 *SmU-boxs* were identified in eggplant, and their expressions under abiotic and biotic stresses were further analyzed. Transcriptomic data analysis revealed the different responses of plant *U-box* gene family members to abiotic stress, indicating functional differences within the family. In addition, *SIU-box13* and *SIU-box40* of tomato have been shown to play a key role in regulating pathogen invasion (Sharma and Taganna, 2020). Therefore, it can be inferred that the *NtU-box* and *SmU-box* genes clustered in the same subfamily with *SIU-box13* and *SIU-box40* may have similar functions. In this study, the expression patterns of 15 selected *NtU-box* genes and 8 *SmU-box* genes under *Ras* infection highlighting their important role in *Ras* resistance. Among the 15 *NtU-box* genes, the expression of 7 *NtU-box* genes (*NtU-box1/3/79/81/82/83/91*) was significantly up-regulated, and the *NtU-box81* gene showed a strong response to *Ras* invasion, which increased more than 8 times at 12 h post-inoculation, while the remaining 8 *NtU-box* genes showed down-regulated expression patterns under *Ras* infection (**Figure 12A**). It is worth noting that certain *NtU-box* genes exhibit contrasting responses to bacterial and drought stresses. For instance, three genes, *NtU-box3*, *NtU-box82* and *NtU-box91* were up-regulated in response to *Ras* infection; whereas they showed down-regulated under drought conditions (**Figures 10A**, **12A**; **Supplementary Table S9**). In eggplant, a similar phenomenon was observed. For example, all 8 selected *SmU-box* genes (*SmU-box1/2/3/23/46/47/48/52*) exhibited up-regulated in response to *Ras* infection (**Figure 12B**), while no significant changes were detected under high temperature stress condition (**Figures 10B**, **12B**; **Supplementary Table S9**). This result suggested a functional differentiation in the tobacco *NtU-box* gene family in their responses to different stresses. Further study of these genes would greatly enhance the understanding of their functions in both tobacco and eggplant.

## Conclusions

In this study, a total of 116 *NtU-box* genes and 56 *SmU-box* genes were identified in the genome of tobacco and eggplant, which were categorized into 5 subfamilies, respectively. These *NtU-box* genes and *SmU-box* genes were randomly distributed on the 24 chromosomes of tobacco and the 12 chromosomes of eggplant. Phylogenetic analysis suggested a shared ancestor predating the divergence of six species (tobacco, eggplant, potato, tomato, *Arabidopsis thaliana*, and pepper), and segmental duplication event was the primary driver for the expansion of the *U-box* gene family in both tobacco and eggplant. The promoters of *NtU-box* and *SmU-box* genes contained *cis*-regulatory elements associated with cell development, plant hormone response, photoresponsive elements, and stress response. The expression levels of the tobacco and eggplant *U-box* genes varied under various abiotic stress conditions. qRT-PCR analysis revealed that 15 selected *NtU-box* and 8 *SmU-box* genes play important roles in response to pathogen *Ras* invasion in tobacco and eggplant. Our results provided valuable information for further functional study of *U-box* genes in both tobacco and eggplant.

## Data availability statement

The datasets presented in this study can be found in online repositories. The names of the repository/repositories and accession number(s) can be found in the article/**Supplementary Material**.

## Author contributions

RC: Writing – original draft. BZ: Writing – original draft, Data curation. GG: Writing – original draft, Resources. CD: Writing – review & editing, Resources. XL: Writing – review & editing, Resources. WC: Writing – review & editing, Resources. YZ: Writing – review & editing, Resources. TL: Writing – review & editing, Data curation. RW: Writing – review & editing, Data curation. XX: Writing – review & editing, Validation.
