# Supplementary figures and images for: Genome-wide identification and expression analysis of the U-box E3 ubiquitin ligase gene family related to bacterial wilt resistance in tobacco (Nicotiana tabacum L.) and eggplant (Solanum melongena L.)
Source: Front Plant Sci. 2024 Jul 30;15:1425651. doi: 10.3389/fpls.2024.1425651 (PMC11319268; doi:10.3389/fpls.2024.1425651)

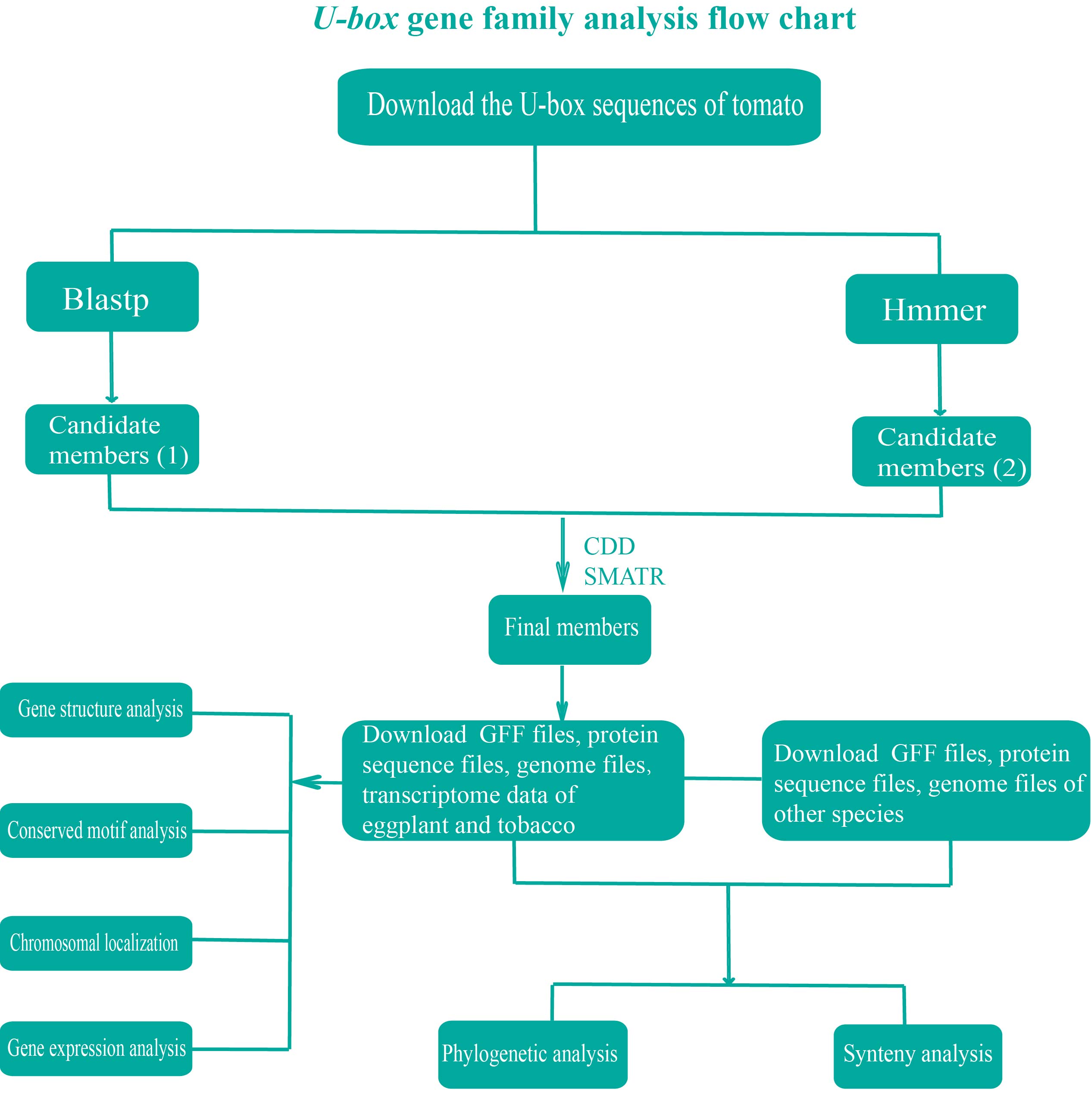

Supplement: Supplementary file 1 [file Image_1.jpeg]

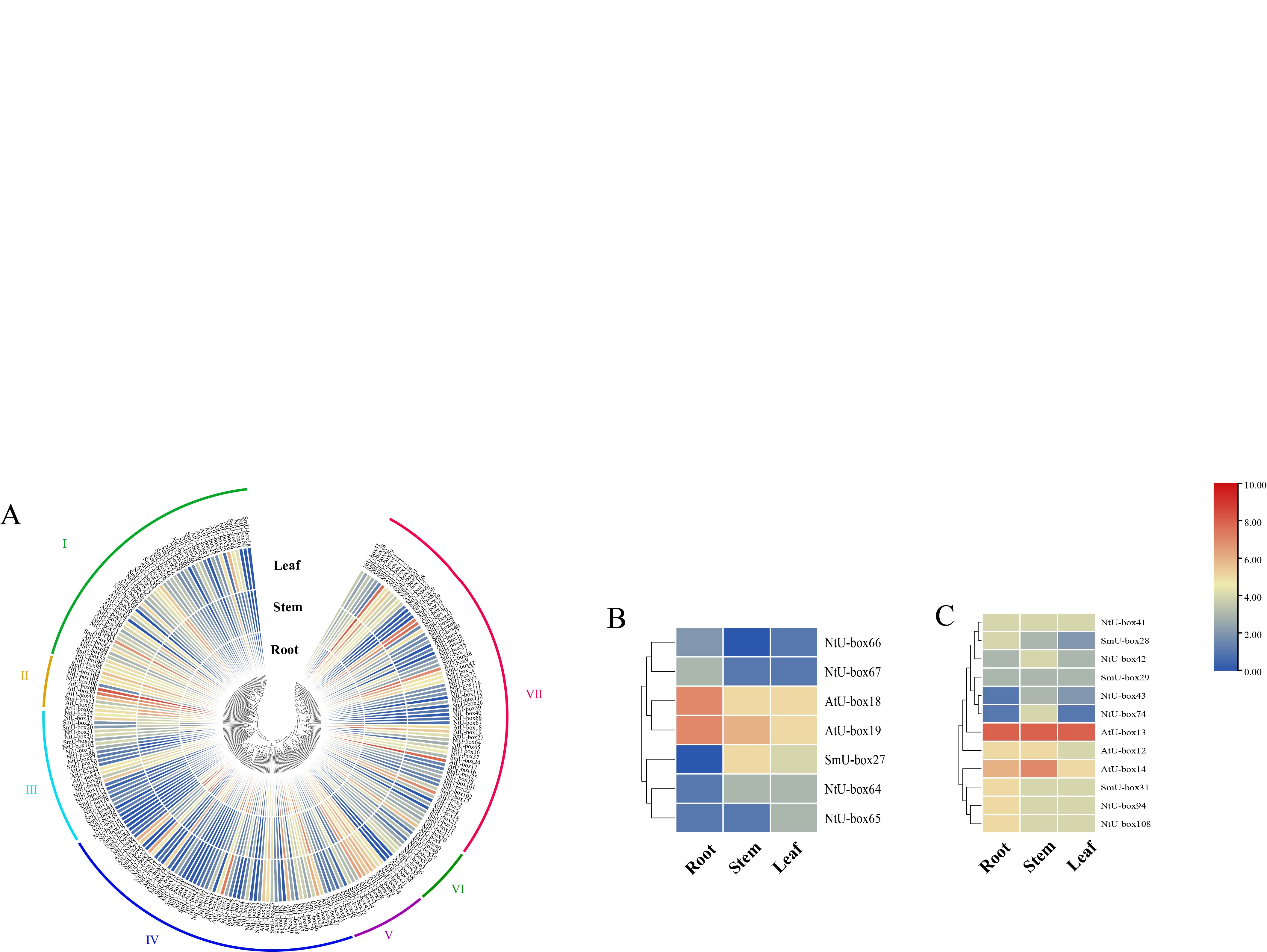

Supplement: Supplementary file 2 [file Image_2.jpeg]
