# Supplementary material for: Genome-wide identification and expression analysis of the U-box E3 ubiquitin ligase gene family related to bacterial wilt resistance in tobacco (Nicotiana tabacum L.) and eggplant (Solanum melongena L.)
Source: Front Plant Sci. 2024 Jul 30;15:1425651. doi: 10.3389/fpls.2024.1425651 (PMC11319268; doi:10.3389/fpls.2024.1425651)
Supplement: Supplementary file 7 [file Table_5.docx]

**Table S5 Sequences of 20 predicted motifs of NtU-box proteins**

| **Motif** | **Width** | **Motif Sequence** |
| --- | --- | --- |
| 1 | 22 | MKDPVIVATGQTYERESIZKWL |
| 2 | 32 | EAGHTTCPKTGQVLTHTDLTPNYTLRRLIQQW |
| 3 | 87 | PPJIDVLKTGSMEAKENAAAALFSLSLJDENKVAIGRSGAJPPLVDLLRNGTPRGKKDAATALFNLSIYQGNKARAVRAGAVPTLLR |
| 4 | 148 | SQQATKAALQVLIDTCPWGRNRIKAIESGAVFELIELELDTTEKRVCELILCILAHLCSCADGRAELLKHAAGIAIVSKRILRVSSTADDRAIQILGSISKFSATSEVLQEMLRVGVVSKLCLVJQADCESKLKDKAREILRTHSRVW |
| 5 | 43 | LAKTNMDNRRIIAEAGAIPLLVNLLSSPDSRIQENAVTALLNL |
| 6 | 104 | DPEYQRTGTLTPKSDVYAFGIIJLQLLTARRANGLIHEVERAIESNNLVDLLDKSAGEWPLEEAKELARLALKCCELRRRDRPDLETEVLPLLERLKEVADMAR |
| 7 | 15 | PEIPDEFRCPISLEL |
| 8 | 49 | SQKTSZFQQEVEILSKJRHPHJVTLLGACPEAGCLVYEYMENGSLEDRL |
| 9 | 22 | GSPRAKENAAAILLSLCSGDGR |
| 10 | 44 | YRIAFEVASALAFLHNSKPEPIIHRDLKPANILLDKNFVSKIGD |
| 11 | 22 | SLQVNGTERAKRKAAELLKLLR |
| 12 | 43 | FTFEEIETATDNFSESLKIGEGGYGKVYKGDLDHTTVAIKVLH |
| 13 | 43 | FQEVTREJEKALDVLPLEKLDISDEVKEQVELVRNQFRRAKFR |
| 14 | 22 | NLPLDHSLLTPNHALRSAIZEW |
| 15 | 31 | GGMVDEALAILANLASHQEGKAAIGDAGAVP |
| 16 | 31 | CIANASHGIERIPTPKPPVTKSHVLKLLREA |
| 17 | 32 | TDAVRATVEFLVRKLASGSPEVQRKAAAEJRL |
| 18 | 16 | SINENNKKLIVEAGAV |
| 19 | 54 | HVRPTITAVPTPMGNSIPINZLEDBVVKAYREDVEAQCSEKLJPYKILCKRKKV |
| 20 | 99 | IQALCLLNTALEKAKNILQHCSESSKLYLALTGDVILSRCERSRNLLEQSLGQVQNMVPVSLAAZISZLIAELKGAIFSLDPSEEEAGKVIIELLQQYT |

**Table S5 Sequences of 20 predicted motifs of SmU-box proteins**

| **Motif** | **Width** | | **Motif Sequence** |
| --- | --- | --- | --- |
| 1 | 24 | IPSEFRCPISLELMKDPVTLATGQ | |
| 2 | 64 | NGSMEAKENAAAALFSLSLJDENKVAIGASGAIPPLVDLLRNGTPRGKKDAATALFNLCIYQEN | |
| 3 | 74 | LSSGSIEVQRAAVAEJRLLAKRNMDNRVLIAEAGAIPLLVKLLSSPDSRIQENAVTALLNLSINENNKGLIMEA | |
| 4 | 16 | TYERESIZKWLDSGNN | |
| 5 | 16 | LTPNYALRRLIQEWCE | |
| 6 | 12 | TCPKTKQELTHT | |
| 7 | 76 | KIGEGAYGDVYKGELHHTPVAIKVLHSAGSQRESZFQQEVEILSKJRHPHJVTLLGACPESGCLVYEYMENGSLED | |
| 8 | 22 | LQQSGTERAKRKAREJLKLLRR | |
| 9 | 31 | IPLLVKLLERGSPRAKENAAAJLLSLCKNSR | |
| 10 | 31 | LLMDPEKGMVDEALAILAILATHQEGRAAIG | |
| 11 | 31 | IMRQFHSVTEKLEQALDGJPYEELDISEEVKEQVELVLSQFKRA | |
| 12 | 117 | PTEYKNTDPVGTLAYIDPEYQETGTLTPKSDIYAFGIIJLQLLTARPPLGLVHEVETAIDKGNLVDJLDPSAGDWPLEZAKELAYLALKCCELNSRDRPDLKSEVLPVLEKLKEVAD | |
| 13 | 31 | PLNWQDRIRIVAEVASALIFLHSSKPEPIIH | |
| 14 | 60 | GRAELLNHPAGLAIVSKKILRVSKVGSERGIKILHSISKYSPTPSVLQEMLSLGVVSKLC | |
| 15 | 21 | NASHGVERIPTPRPPVDKSQV | |
| 16 | 141 | YYCKIEGJLKJLKPILEAIVDVEAASSELLQKAFGGLAQFVDELRELCETWZPLCSKVYFVLQAEPLIGKIRTCSLEILELLKSSHKCLRADVTLPSLENCILKIKHVDYELISMTITKVIKAQMEGLGANSDNFAKIADC | |
| 17 | 41 | RPLPESGIQALCKLKKALESAKELLKHCSEGSKJYLALEGE | |
| 18 | 32 | ISQKATKAALHVLIYACPWGRNRVKAIEAGAI | |
| 19 | 84 | KGRVDTPDMELAEDLLVLYSKSNDRAADSASJRRLVEKLQLTTVEDLKQESLALHEMVKERKGDPEEKTEKIVGLLKKIKDFVG | |
| 20 | 21 | DLKPANILLDRNFVSKIGDVG | |
